# A combination of quantitative and qualitative methods in investigating risk factors for lost to follow-up for tuberculosis treatment in Japan – Are physicians and nurses at a particular risk?

**DOI:** 10.1371/journal.pone.0198075

**Published:** 2018-06-15

**Authors:** Lisa Kawatsu, Kazuhiro Uchimura, Akihiro Ohkado, Seiya Kato

**Affiliations:** 1 Department of Epidemiology and Clinical Research, the Research Institute of Tuberculosis, Japan Anti- tuberculosis Association (RIT/JATA), Tokyo, Japan; 2 the Research Institute of Tuberculosis, Japan Anti- tuberculosis Association (RIT/JATA), Tokyo, Japan; Fundació Institut d’Investigació en Ciències de la Salut Germans Trias i Pujol, Universitat Autònoma de Barcelona, SPAIN

## Abstract

**Background:**

The treatment success rate of pulmonary tuberculosis (PTB) patients aged 64 years and below in Japan, a tuberculosis (TB) middle-burden country with a notification of 13.9 per 100,000 populations in 2016, has been fluctuating around 70% for some years. In order to improve treatment outcome, it is critical to address those lost to follow-up (LTFU). The objective of the study therefore was to describe the characteristics of, and analyze the risk factors for those LTFU among pulmonary TB patients aged between 15 and 64, and discuss policy implications.

**Methods:**

The study used a mixed method of quantitative and qualitative approach, and was conducted in two phases. The first involved analysis of cohort data from the national TB surveillance of PTB patients newly notified between 1 January 2006 and 31 December 2015. The second phase involved focus group (FGD) discussions with public health nurses, who are responsible for supporting TB patients’ adherence to medication, on the possible reasons why some patients become lost to follow-up.

**Results:**

Analysis of the surveillance data suggested that among all patients, positive sputum smear (adjusted odds ratio, [aOR] 0.52, 95% confidence interval [CI] 0.47–0.58) and cavitary lesion on chest x-ray (aOR 0.79, 95%CI 0.72–0.85) decreased the risk, while not requiring hospitalization increased the risk of LTFU (aOR 1.46, 95%CI 1.33–1.60). Among females, being a physician (aOR 2.07 95%CI 1.23–3.48) and nurse (aOR 1.18, 95%CI 1.91–1.37) were identified as additional risk factors for LTFU. The analysis of focus group discussions revealed three possible themes which may be useful in understanding why nurses and physicians were at a higher risk of becoming LTFU–firstly, the possibility that physicians and nurses were finding it difficult to make medication taking a routine, secondly, their low risk perception towards TB is affecting their adherence behavior, and thirdly, their unwillingness to accept DOTS was increasing their risk of becoming LTFU.

**Conclusions:**

The analysis of surveillance data and FGD transcripts indicated that patient education for those starting their treatment as an outpatient, and establishing DOTS that is both acceptable and realistic to physicians and nurses, may be two issues which need to be addressed urgently.

## Background

Japan is a tuberculosis (TB) mid-burden country, with a notification of 13.9 per 100,000 population in 2016 [1]. One of the unique characteristics of TB epidemiology in Japan is its aging patients–currently, approximately two-third and one-third of the patients are aged 65 years and above and 80 years and above, respectively [1]. The proportion of died among the treatment outcome of TB patients in Japan is therefore quite significant, which subsequently is suppressing the proportion of treatment success–among the total number of 14,123 pulmonary TB patients (PTB) newly notified in 2015, 17.0% had died while only 52.9% had successfully completed treatment [1].

However, even among the PTB patients notified in 2015 who were aged 64 and below, the proportion of treatment success was 69.3% [1]. There is still some room for further improving treatment results, and one way is to address those lost to follow-up (LTFU). It has also been reported that among patients retreated due to LTFU from initial treatment, drug resistance is more common and retreatment outcomes inferior [2,3]. In Japan, reports have been published on treatment outcome of the general population^1^, as well as among specific populations such as foreign-born patients [4] and patients with homeless history [5], however, no study has so far analyzed the national TB surveillance data to examine specifically those who were LTFU. Our aim was thus to describe the characteristics of, and analyze the risk factors for those LTFU among pulmonary TB patients aged between 15 and 64, and discuss policy implications.

## Methods

The study was conducted in two phases. The first phase was conducted between February and April 2017, and consisted of a cross-sectional study whereby aggregated cohort data from the Japan TB Surveillance (JTBS) of pulmonary TB patients aged between 15 and 64 and newly notified between 1 January 2006 and 31 December 2015 was analyzed. Under the JTBS, treatment outcome of pulmonary TB patients is evaluated at the end of 12 months. The outcome categories include “cured”, “treatment completed”, “died”, “treatment failure”, “lost to follow-up”, “transferred out”, “still on treatment”, and “unevaluated”. The outcome categories are automatically calculated by the internal algorithm of the JTBS, which in principle follow the WHO guideline [6]. For the purpose of our study, “cured” and “treatment completed” were collapsed into “treatment success”. According to JTBS, a patient is deemed LTFU if he or she has interrupted treatment for two consecutive months or longer, or, his or her treatment duration falls short of standard treatment duration. The definitions of all other outcomes can be found in S1 File.

Those LTFU were extracted and analyzed in terms of sex and age groups. Those who did not receive treatment, and whose treatment status was unknown, were excluded from the study population. Multiple-regression analysis was conducted with LTFU as independent variable, and the following as dependent variables–sex, country of birth (Japan- or foreign-born), job category and status, history of homelessness in the previous one year, cavitary lesions on chest x-ray (CXR), sputum smear examination results, being on welfare scheme, not being under any health insurance and initial hospitalization. The variables were chosen based on previous literatures [7,8,9, 10]. HIV status and multi-drug resistance, although often indicated as a risk factor, were not included, as the number of patients is extremely small in Japan–in 2015, among the 17,625 newly notified TB patients, 44 (0.2%) were notified as HIV positive, and among the 11,151 culture confirmed TB cases, 50 (0.4%) had multidrug resistance^1^. Analyses were conducted for all patients, then further sub analyses by sex. R version 3.1.3 (R Development Core Team, Vienna, Austria) was used for all statistical analyses.

The second phase involved focus group discussions (FGD) with public health nurses working at public health centers, who are responsible for overseeing adherence support for TB patients, including organizing and conducting DOTS, in Japan. Under the so-called “Japan DOTS” strategy, sputum smear positive patients are hospitalized for at least two months, until they are considered no longer infectious, and during which they receive intensive hospital DOTS. Upon discharge, they, as well as non-infectious patients, are assessed of their risk of interrupting the treatment by local public health nurse using a risk assessment form, and are categorized as requiring any one of the three types of adherence support–type A, consisting of directly observed therapy every day, type B, consisting of adherence support once to twice a week, and type C, once to twice a month.

The FGDs were conducted with the purpose of further exploring one particular aspect of the results from the analysis conducted in the first phase, which indicated that physicians and nurses were at a risk from becoming LTFU. The FGDs were organized in September 2017, with public health nurses who were attending a training course on TB at the Research Institute of Tuberculosis, Tokyo, Japan. Upon orientation to the course, all participants were introduced to the study, and invited to take part in a FGD. 13 participants gave written consent to participate, and were divided in two groups. The participants were first asked to discuss on their general experience of patients who were, and whom they felt were at a high risk of becoming LTFU. In the latter part of the discussion, the participants were shown the results of the analysis of the TB surveillance data on treatment outcomes, and were asked to exchange their thoughts, with a particular focus on assisting physicians and nurses in treatment adherence. Each discussion lasted approximately 60 minutes, and both discussions were voice recorded, upon consent of all participants, using an integrated circuit recorder and were transcribed verbatim.

The FGD data were analyzed by performing interpretive content analysis, which firstly involved familiarization of the text through review of all transcripts. A thematic framework was then designed using an iterative process, and segments describing the perceived risk factors for LTFU were retained for further analysis. These segments were then organized by means of open coding and abstraction, to see if any new discourses arose, which could help us further understand why physicians and nurses were at a higher risk of becoming LTFU.

The study protocol was approved by the Ethical Committee of the Research Institute of Tuberculosis, Japan Anti-tuberculosis Association (reference number: RIT/IRB 29–1).

## Results of the analysis of surveillance data

### General trend in the treatment outcomes

**Fig 1:**
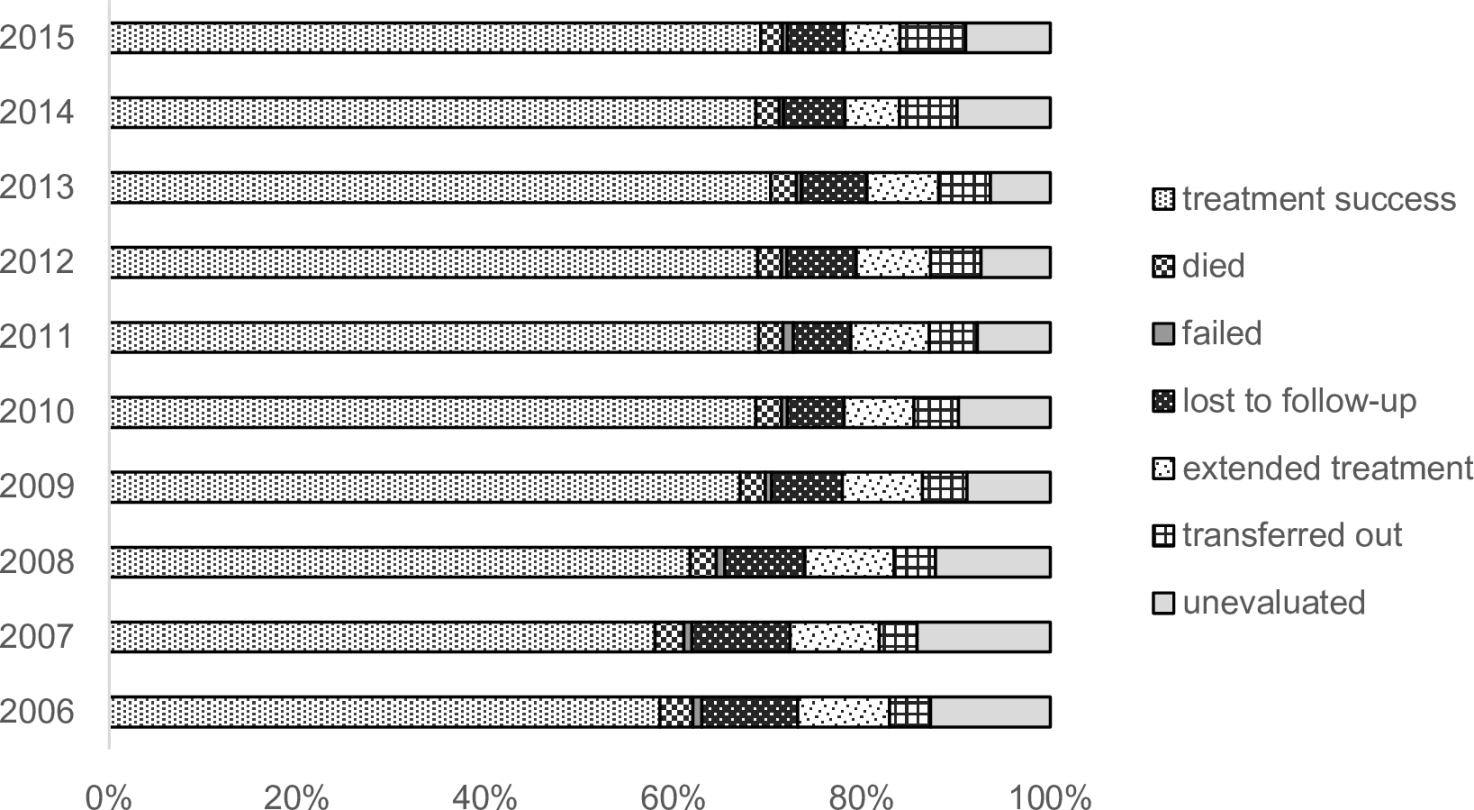
Treatment outcome of pulmonary TB patients aged 15 to 64 years old, notified between 2006 and 2015.

Between 2006 and 2015, a cumulative total of 175,222 PTB patients were notified to the JTBS, out of which 73,591 were aged 15 to 64. Treatment outcome of PTB patients aged 15 to 64 years notified between 2006 and 2015 is summarized in Fig 1 (see also S1 Table). Although the proportion of treatment success has gradually improved over time, this has been largely attributable to the decrease in those whose treatment outcome was unevaluated and those still on treatment. The proportion of those LTFU has decreased from 10.2% in 2006 to 6.1% in 2011, however, since then, has shown a modest increase.

### Characteristics of those lost to follow-up

Over the study period, of the cumulative total of 73,591 patients, 5,760 (7.8%) had been lost to follow-up. The general characteristics of those LTFU are summarized in Table 1.

**Table 1:** Characteristics of those LTFU aged 15 to 64 years, in Japan, 2006–2015.

	n	%		n	%
**TOTAL**	**5,760**	**100**	**Job category**		
**Sex**			Full- & part-time	2,633	45.7
Male	3,552	61.7	Temporary & self-employed	718	12.5
Female	2,208	38.3	Unemployed	1,166	20.2
**Age groups (years)**			Physicians	50	0.9
15–24	598	10.4	Nurses	285	4.9
25–34	1,219	21.2	Other healthcare workers	146	2.5
35–44	1,231	21.4	High-school & university students	335	5.8
45–54	1,096	19.0	Housemakers	239	4.1
55–64	1,616	28.1	Unknown	188	3.3
**Country of birth**			**TB Treatment history**		
Japan-born	5,045	87.6	New	5,455	94.7
Foreign-born	568	9.9	Retreatment	284	4.9
Unknown	147	2.6	Unknown	21	0.4

LTFU: lost to follow-up

TB: tuberculosis

**Fig 2:**
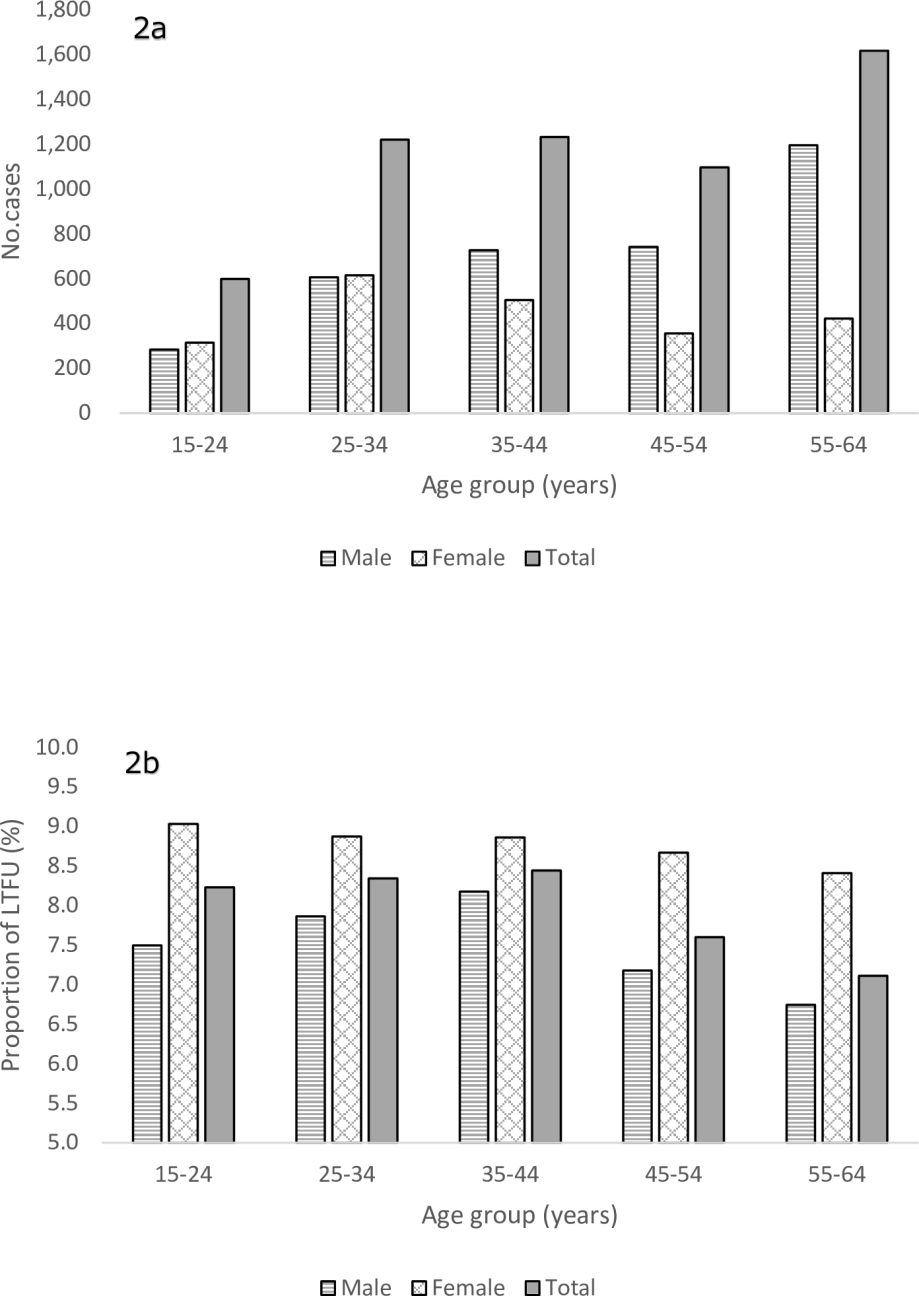
Number and proportion of patients lost to follow-up, by sex and age groups, 2006–2015. (a) Number of patients lost to follow-up, by age and sex groups. (b) Proportion of patients lost to follow-up, by age and sex groups.

The majority was Japan-born and with no history of previous TB treatment. 61.7% (n = 3,552) of the LTFU patients were males, with the largest number in those aged 55 to 64 years old. However, among the females, the largest number was found in those aged 25 to 34 years old (Fig 2A). Furthermore, in all age groups, the proportions of those LTFU out of all pulmonary TB patients were higher among females than males, with the overall proportion of LTFU at 8.8% among females and 7.3% among males (Fig 2B). The highest proportion of LTFU was found among females aged 15 to 24 years old (9.0%, 314/3,477), followed by females aged 25 to 34 and 35 to 44 years old (8.9%, 614/6,918 and 504/5,687) (see also S2 Table).

**Fig 3:**
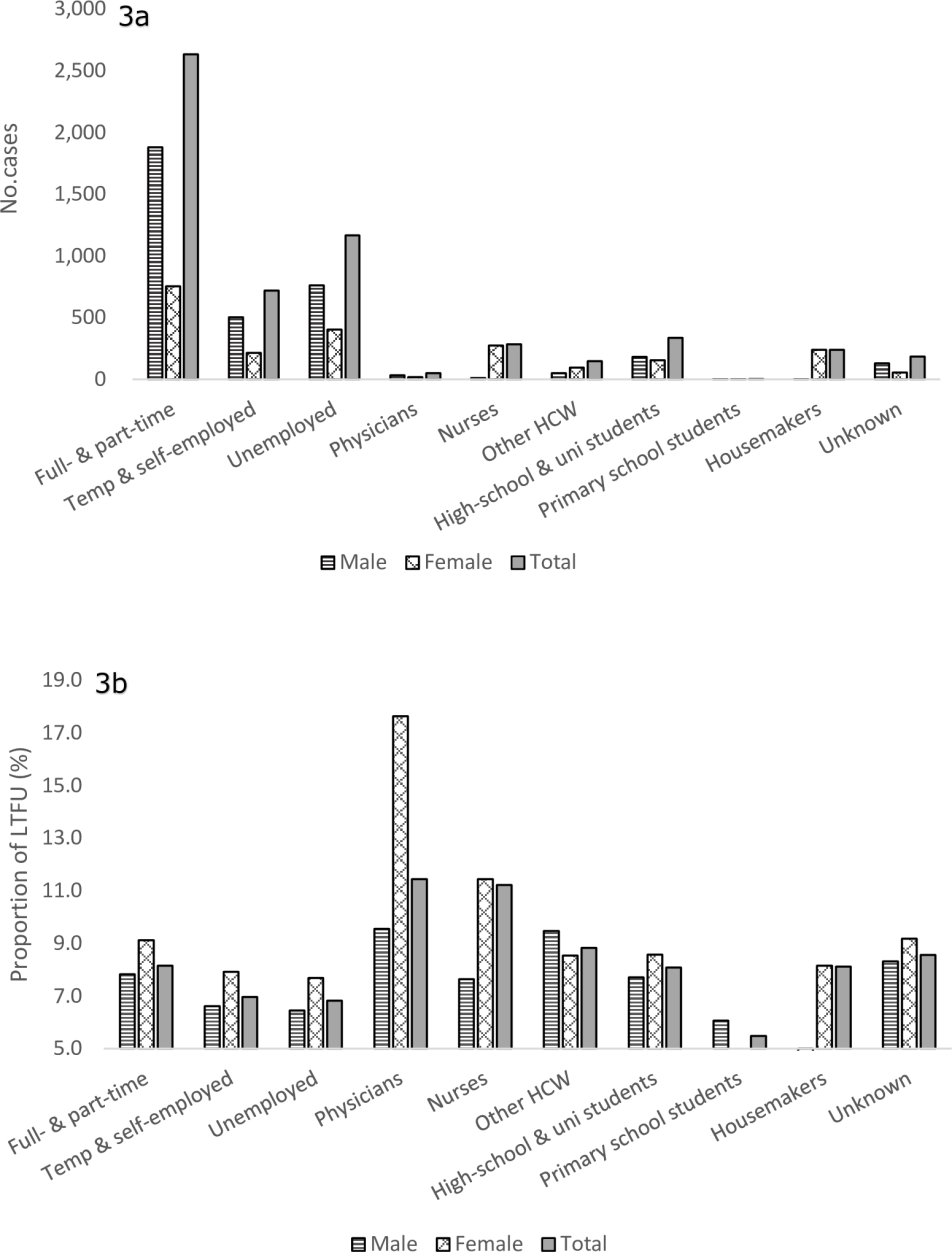
Number and proportion of patients lost to follow-up, by job status and job categories, 2006–2015. (a) Number of patients lost to follow-up, by job status and job categories. (b) Proportion of patients lost to follow-up, by job status and job categories.

Looking at those LTFU by their job status and categories, the largest number of LTFU patients were diagnosed among those with full-time and part-time workers among both males and females, followed unemployed and/or temporary or self-employed workers for males, and nurses among females (Fig 3A). However, though the proportions of LTFU were relatively similar across the different job categories among males, a significantly higher proportion of LTFU was observed among physicians and nurses among females (17.6%, 18/102 and 11.4%, 274/2,394) (Fig 3B, see also S3 Table).

### Risk factors to lost to follow-up

**Fig 4:**
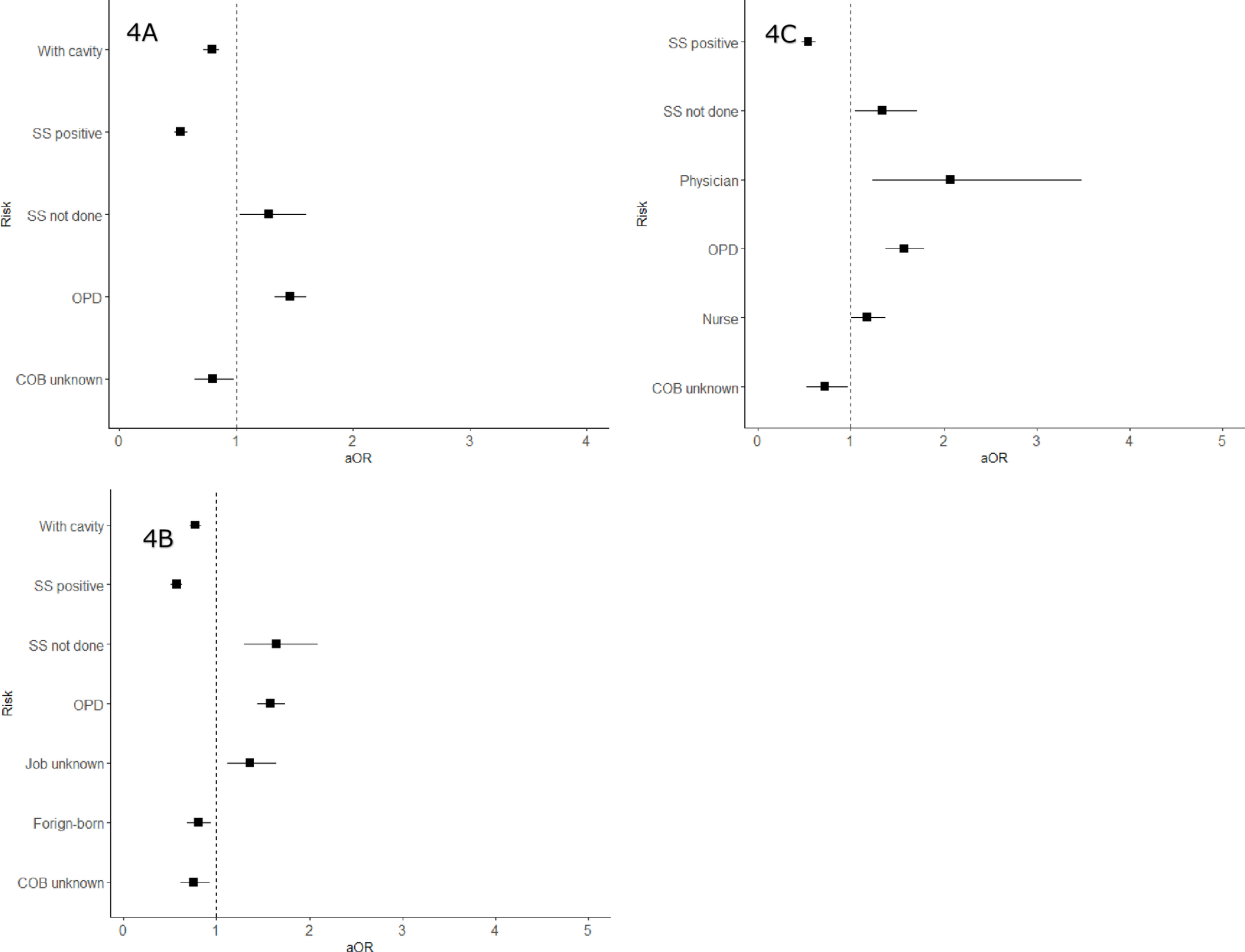
Forest plot showing odds ratios for selected risk factors. (a) Risk factors identified as significant for all patients (b) Risk factors identified as significant for males (c) Risk factors identified as significant for females. SS: sputum smear, OPD: outpatient department, COB: country of birth, aOR: adjusted odds ratio.

Fig 4A–4C are the forest plot of adjusted odds ratios of factors for LTFU which were significant for all patients, males and females respectively. The full result of the multiple regression analysis can be found in S4 Table. Among all patients, positive sputum smear (adjusted odds ratio, [aOR] 0.52, 95% confidence interval [CI] 0.47–0.58), cavitary lesion on CXR (aOR 0.79, 95%CI 0.72–0.85), and country of birth unknown (aOR 0.80, 95%CI 0.64–0.98) were protective factors for LTFU. On the other hand, not requiring hospitalization, in other words, being an outpatient from the start of the treatment, was a risk factor for LTFU (aOR 1.46, 95%CI 1.33–1.60), as well as spuum smear not done (aOR 1.28, 95%CI 1.03–1.60), being a nurse (aOR 1.22, 95%CI 1.03–1.44) and job category unknown (aOR 1.34, 95%CI 1.08–1.66). The results were relatively similar when the analysis was conducted among the male patients only, with positive sputum smear (aOR 0.57, 95%CI 0.51–0.63), cavitary disease (aOR 0.77, 95%CI 0.71–0.84), being foreign-born (aOR 0.81, 95%CI 0.69–0.94) and country of birth unknown (aOR 0.75, 95%CI 0.61–0.93) identified as protective factors, and not requiring hospitalization (aOR 1.58, 95%CI 1.44–1.74), smear not done (aOR 1.65, 95%CI 1.30–2.09), and job category unknown (aOR 1.36%CI 1.12–1.65) as risk factor. However, among the female patients, while positive smear (aOR 0.54 95%CI 0.47–0.62) and country of birth unknown (aOR 0.72 95%CI 0.53–0.97) were similarly identified as protective factors, and not being hospitalized (aOR1.57 95%CI 1.37–1.79), smear not done (aOR 1.34, 95%CI 1.04–1.71) similarly as risk factors, two job categories, namely physician (aOR 2.07 95%CI 1.23–3.48) and nurse (aOR 1.18, 95%CI 1.91–1.37) were also identified as risk factors for LTFU.

### Results of the analysis of FGDs

In exploring the possible reasons why physicians and nurses were at a higher risk of becoming LTFU, three broad themes were identified. These were termed as “difficulty in making medication taking a routine”, “low risk perception”, and “unwillingness to accept adherence support”. Each is elaborated in detail below.

### Difficulty in making medication taking a routine

A number of participants agreed that they felt that it was difficult for nurses and physicians to make medication taking a routine practice, simply because of the nature of the work;

“…because they (physicians and nurses) have nightshifts… it seems difficult for them to set a particular time of the day to take medicines.”

“Their work is irregular and really tough. Sometimes they’re too tired to take the medication and forget. Of course, they forget once, then it’s easy to forget the next time, and next time…”

The above two comments were regarding a physician and a nurse on TB treatment, however, a similar opinion was voiced regarding a physician on LTBI treatment.

“My current patient…she is a young female physician, and she’s only just started (her treatment) but already, she’s told me she’s not sure whether she can complete it. Like, she decides to take the medication in the morning, but then she has nightshift, then there is no morning for her…”

Although not all physician and nurse patients whom the participants had assisted had become LTFU, several experienced treatment interruptions and occasions whereby their patients admitted forgetting to take their medication “because they were busy”.

### Low risk perception

Low risk perception arising from familiarity, especially on behalf of the physicians, was voiced out by several participants. The participants had felt that this in turn affected the motivation and attitude of the physicians towards TB medication. The following excerpt illustrates this concern;

“I think the physicians are too used to seeing diseases… like, it’s not a special event for them.”

“I agree…I don’t think they’re too worried (about them getting TB). So they…may not be so serious about the treatment.”

“Or …because they take (other) medications quite casually…TB medicine is no different…”

The last quote expresses the participant’s concern that because physicians may regard TB medication no different from other medications, they may be less serious about the consequences of interrupting TB treatment. Another participant commented that the way physicians make estimates about the dangerousness of health risks appear to be totally different from “ordinary” people.

“Like needle-stick injury…if we (public health nurses) ever get ourselves involved in a needle-stick injury, it’s a big matter, right? But for physicians, it’s nothing! I once assisted a physician…can’t remember exactly, but it involved administering an anesthetic to a patient, and the physician was…pricking himself all over his fingers, as if it were nothing…so may be, I thought, if he ever gets TB, he will probably think, or not think, about the various consequences the same way he did with needle pricks.”

### Unwillingness to accept adherence support

Finally, a number of participants elaborated their experience with physicians and nurses who were not willing to accept adherence support, and of struggling to build a relationship of trust.

“I had one nurse patient…who started off by saying ‘you don’t have to tell me about DOTS (directly observed treatment, short-course), I know all about it’. But the next thing she said was, she didn’t want it (i.e. didn’t want to be checked up by the public health nurse on a regular basis). So we came to a compromise, I call up her once a month…but she doesn’t pick up her phone. It becomes once every one and a-half month… then every two months…it’s no more DOTS.”

“I am currently supposed to be assisting one physician. But every time I call him up to check if he’s taking medications alright, he is like ‘yes, yes, everything’s fine’ and hangs up the phone. Then I can’t help but think, even if he has forgotten to take his medication, he will never tell me the truth”

“Oh it’s so difficult to get them (physicians) on DOTS…they’re professionals. Why should they be checked up by us, the public health nurses? I think that’s what they think.”

“I really find it hard to build a relationship with physicians…they have the knowledge, so they think they don’t need us giving them advices …rather than asking us questions, they would much rather consult their colleagues”.

In fact, the difficulty in communicating with physicians and nurses were experienced not only in the TB patient-public health nurse relationship, but also in other moments, such as public health nurses attending a DOTS meeting at a hospital, or calling the hospital to confirm patient information, or to ask for results of certain examinations.

Gender difference, in other words the possible reasons why the risk was particularly high among female physicians and nurses, could not be explained by the participants.

## Discussion

To our knowledge, ours is the first study to have examined specifically the risk factors for LTFU, using national TB surveillance data, in Japan. Social and demographic factors such as male gender [7,8], foreign nationality [9,10] and unemployment [11, 12] and treatment- and condition-related factors such as history of previous TB treatment [13,14] and positive sputum smear [13,15], which have often been reported as predicting LTFU, were not significant factors in our study. This to a large extent can be explained by the DOTS strategy unique to Japan, which has been developed and implemented since 2000. The risks assessed include those factors mentioned earlier–hence our results which, although seemingly contradict the findings of earlier studies, are in fact a natural consequence of the “Japan DOTS” strategy with intensified adherence support for those considered as with risk to LTFU.

In our study, positive sputum smear and cavitary lesions were even identified as protective factors (cavitary lesions was not significant among females but the odds ratio was in the same direction as males)–these may be considered as a proxy of perceived severity of disease. Indeed, several studies from abroad have reported that lack of symptoms and patients feeling better were associated with poorer compliance [9,16]. On the other hand, being an outpatient from the start of the treatment was associated with the risk of LTFU, independent of the severity of the disease. This potentially indicates the important role of hospitalization in patient education in Japan i.e. the so-called hospital DOTS. The critical importance of appropriate and adequate health education and counselling in preventing LTFU has long been stressed, and repeatedly confirmed across numerous countries of differing TB burden [7,8,17,18]–our results indicate that current opportunities for patient education for outpatients may not be sufficient, and thus putting them at a higher risk to LTFU than hospitalized patients.

Another interesting, and potentially a worrying finding was that being a physician or a nurse was identified as a risk factor among females. Reports on the effect of occupation to LTFU are in fact very limited–one study from Malaysia has similarly examined the effect of occupation to LTFU, however, the authors did not find any statistically significant results [13]. On the other hand, several studies from the US have indicated poor adherence among healthcare workers in seeking preventive therapy for tuberculosis [19], and poor treatment completion rates among those with active TB [20]. However, the studies mentioned above do not discuss in details why healthcare workers have performed poorly. Outside the field of TB, non- or poor- compliance with medication and other health and hygiene behaviors among healthcare professionals have also been reported [21–24]. Due to the relatively small sample size, we were not able to conduct sub-analyses by, for example, specialty or years of post-internship experience, and thus could not identify possible reasons behind poor compliance among the medical doctors from the quantitative data. The analysis of the FGDs, however, have to a certain extent shed some light–namely, the possibility that physicians and nurses finding it difficult to make medication taking a routine, that their low risk perception towards TB is affecting their adherence behavior, and that their unwillingness to accept DOTS is increasing their risk of becoming LTFU.

One possible solution, which was also identified by several of the participants of the FGDs, may be the use of infection control nurses in the hospitals to ensure adherence to TB treatment among nurses and physicians. By doing so, the public health nurses were able to achieve two objectives; firstly, they could avoid contacting the patients directly, an action which was apparently unwelcomed by physicians and nurses. Secondly, by involving infection control nurses, DOTS became a part of an infection control measurement, and not a mechanism to “check-up” the individual act of taking medication (which was again unwelcomed by physicians and nurses). Compliance with an infection control measurement may be more easily acceptable as a professional and official duty, and the consequences of non-compliance recognized as being more far-reaching, than with one’s TB treatment.

Lastly, though the level of significance varied across males and females, the odds ratios were in the same direction to point that unknown job category, sputum smear status unknown and sputum smear not done were a risk factor for LTFU. This may be due to the job category and sputum smear status “unknown” resulting from public health nurse not being able to contact the patient to conduct adequate epidemiological investigation. As explained in the Methods, although initial defaulters (those who did not start treatment) were excluded from the analysis, it is possible that patients whose job category is unknown are similar to initial defaulters in nature, and are therefore at a higher risk of interrupting the treatment. Sputum smear status “not done” may be an indication of a slightly more serious issue–the proportion of “examination not done” among the females was twice that of males (2.3% vs 1.2%). Exploration of the reasons as to why females have higher proportion of sputum examination “not done” is beyond the scope of this study–however, it has been previously indicated that females face greater cultural and social inhibitions about producing deep sputum compared with males [25].

Our study had several limitations. Firstly, as we analyzed the data from surveillance, factors not included in the JTBS, such as smoking and alcohol which are occasionally indicated as risk factors to LTFU, could not be examined. On the other hand, as mentioned above, these factors are already included in the risk assessment form used by public health nurses in Japan. Policy implications would therefore have been minimal, even if these factors had been included in the analysis. Secondly, timing of LTFU was not considered in our study. Interventions to prevent LTFU may need to be tailor-made according to the different timings at which LTFU occur. Thirdly, examination of the possible gender aspect of LTFU remained minimal, due to limited data available from the JTBS. Lastly, the FGDs were conducted with public health nurses, and not the patients themselves. The possible reasons for LTFU were thus identified from the perspective of health service provider, which may differ from patient perspective. A similar qualitative study with patients may provide additional insight to understanding LTFU.

## Conclusions

A combined methodology of analysis of surveillance data and FGD transcripts revealed several potential risk factors for LTFU. The results indicated that patient education for those starting their treatment as an outpatient, and establishing DOTS that is both acceptable and realistic to physicians and nurses, may be two issues which need to be addressed urgently.

## Supporting information

S1 File Definitions of treatment outcomes under the Japan tuberculosis surveillance system. (DOCX)

S1 Table Treatment outcome of pulmonary TB patients aged 15 to 64 years old, notified between 2006 and 2015. (XLSX)

S2 Table Number and proportion of patients lost to follow-up, by sex and age groups, 2006–2015. (XLSX)

S3 Table Number and proportion of patients lost to follow-up, by job status and job categories, 2006–2015. (XLSX)

S4 Table Crude and adjusted odds ratios of risk factors for lost to follow-up. (XLSX)
