# Supplementary material for: A combination of quantitative and qualitative methods in investigating risk factors for lost to follow-up for tuberculosis treatment in Japan – Are physicians and nurses at a particular risk?
Source: PLoS One. 2018 Jun 15;13(6):e0198075. doi: 10.1371/journal.pone.0198075 (PMC6003677; doi:10.1371/journal.pone.0198075)
Supplement: S1 File — (DOCX) [file pone.0198075.s001.docx]

**Treatment outcomes of drug susceptible TB**

Cohort classification has undergone a major revision in 2007, and the definitions of the treatment outcomes currently used in Japan are as follows;

Cure: A pulmonary TB patient who has undergone treatment of sufficient duration (no longer than 12 months), who was culture-negative on two consecutive occasions, with one within three months of treatment completion and another on previous occasion.

Completed treatment: A pulmonary TB patient who has undergone treatment of sufficient duration (no longer than 12 months) and either whose last culture test was negative on at least one occasion, or whose culture test result could not confirmed.

Died: A pulmonary TB patient who has died from any cause during treatment.

Failure: A pulmonary TB patient whose culture test was positive at month 5 or later during treatment.

Lost to follow-up: A pulmonary TB patient whose treatment was interrupted for 60 consecutive days or more, or for two consecutive months, or whose treatment duration is deemed insufficient.

Transferred out: A pulmonary TB patient who has moved out of the catchment area of a public health center during treatment.

Still on treatment: A pulmonary TB patient who is still on treatment at month 12.

Not evaluated: A pulmonary TB patient whose treatment outcome could not be evaluated for one or more of the following reasons – the patient did not start treatment or has died prior to starting the treatment, the treatment regimen was unknown, the treatment regimen was not the standard regimen, either isoniazid, rifampicin or both were discontinued during the treatment.
